# Mercury Contamination and Co-exposures in the Amazon Basin: At the Center of the Planetary Environmental Crisis

**DOI:** 10.5334/aogh.4817

**Published:** 2025-07-29

**Authors:** Roberto G Lucchini, Paulo Cesar Basta, Maria Elena Crespo-Lopez, Maria del Carmen Gastañaga, Cristina O’Callaghan-Gordo, Jesus Olivero-Verbel, Claudia Vega, Stefanny Magaly Moncada Barbosa, Carlos Espinal, Quentin Felty, Alok Deoraj

**Affiliations:** 1 School of Public Health and Social Work, Florida International University, Miami, USA; 2 Occupational Medicine, University of Modena, Italy; 3 Escola Nacional de Saúde Pública (ENSP), Fundação Oswaldo Cruz (Fiocruz), Rio de Janeiro, Brasil; 4 Laboratorio de Farmacología Molecular, Universidade Federal do Pará (UFPA), Belem, Brasil; 5 Centro Nacional de Salud Ocupacional y Protección del Ambiente para la Salud. Instituto Nacional de Salud, Lima, Perú; 6 Barcelona InTerdisciplinary research group on plAnetary heaLth (BITAL), Faculty of Health Sciences, Universitat Oberta de Catalunya, ISGlobal, Universitat Pompeu Fabra, Barcelona, Spain, CIBERESP, ISCIII; 7 Environmental and Computational Chemistry Group. Research Institute on Climate Change and Sustainable Development. School of Pharmaceutical Sciences, Universidad de Cartagena, Colombia; 8 Centro de Innovación Científica Amazónica CINCIA, Puerto Maldonado, Perú; 9 Departamento de Gobierno, Universidad del Desarrollo, Las Condes, Región Metropolitana, Chile

**Keywords:** amazon, mercury, mining, indigenous health, inequalities, agrochemicals, microplastics

## Abstract

*Background:* Mercury contamination remains a significant public health concern in the Amazon basin. This review synthesizes recent evidence on mercury exposure, health outcomes, and emerging co-exposures in the Amazon countries. Data were presented at the Annual Conference of Global Health in the Americas, organized by Florida International University in Cartagena, Colombia on September 15, 2023, at a virtual session of the Consortium of Universities for Global Health on November 29, 2023, and subsequently updated with further literature search.

*Findings:* Reported mercury concentrations in fish range from 0.10 to 4.73 µg/g, while hair mercury levels in exposed populations span 3.07–24.6 µg/g. Cross-sectional studies among Indigenous and traditional communities consistently demonstrate associations between mercury biomarkers—primarily measured in hair and urine—and neurocognitive as well as neuromotor impairments. Additional evidence links mercury exposure to increased cardiovascular and metabolic risk. Genetic susceptibility, notably APOE4 and GSTP1 polymorphisms, may modulate mercury absorption and toxicity. Co-exposures to microplastics and agrochemicals are increasingly reported in the region, raising concern over synergistic toxic effects. However, scientific evidence on these combined exposures remains fragmented and insufficient.

*Conclusions and recommendations:* To address this critical gap, we propose the formation of a cross-national scientific consortium to foster collaboration, enhance epidemiological capacity, and strengthen laboratory infrastructure. Crucially, efforts to address mercury contamination must center the voices of Amazonian Indigenous peoples, who bear the greatest burden of exposure while facing persistent social, environmental, and health inequalities. Meaningful engagement with these communities is essential to overcome marginalization and ensure that research, policy, and intervention strategies are culturally informed, equitable, and effective. Coordinated regional action is urgently needed to protect the health and rights of vulnerable Amazonian populations.

## 1. Introduction

As pointed out in a recent editorial of *Science*, the two parallel processes of deforestation and degradation from timber and gold extraction, and habitat fragmentation, fires, and drought in the Amazon are proceeding much faster than previous environmental changes [1]. This environmental crisis impacts quite dramatically the health of millions of people and needs to become an urgent priority of the local and global agenda [2]. The gold market is an especially relevant driver in this context because the extraction of this mineral in the Amazon requires intensive use of mercury. The high increase in gold price in the last 20 years [3] reached 3,500 USD/Oz in April 2025, a value that exceeds all the predictions and is expected to keep rising. The increase is even higher considering the devaluation of the national currencies of the Amazonian countries in relation to the US Dollar. This creates a strong demand, especially from areas like the Amazon, where extraction and production costs are significantly reduced compared to the major world producers—China, Russia, Australia, Canada, and the USA [3]. Artisanal and small-scale gold mining (ASGM) is recognized as the primary anthropogenic source of airborne mercury emission in the Amazon, although the term “artisanal and small-scale” can hardly be applied to what is currently happening in this region [4]. Brazil and Peru are among the leading countries responsible for it because 80% of South American emissions originate from the Amazon [5]. Nevertheless, a more accurate approach is needed to understand the complexity of mercury contamination [4]. Mining is related to artisanal and small-scale activities but also to larger operations with heavier and more expensive machinery. Besides mercury, mining causes contamination of the sediments and water with many other toxic metals in the entire Amazon basin [6]. Besides mining, environmental contamination is also increased due to land use changes such as accelerated deforestation for soy and beef production and land use for energy-generating dam construction [7]. Oil extraction in the Peruvian Amazon contributes to generating contamination and human exposure to other toxic metals like lead, arsenic, and cadmium in addition to mercury [8]. A tipping point of the environmental health crisis is soon to be reached, turning the Amazon into a *degraded ecosystem,* as recognized at the high-level summit held in Belém, Brazil, in August 2023. The nine Amazonian countries of Brazil, Peru, Colombia, Bolivia, Ecuador, Venezuela, French Guiana, Guyana, and Suriname agreed on a protection plan for the forest and support for the Indigenous People, as reported in the Belem Declaration [9]. Notably, the Declaration specifically indicates the need for research-based recommendations, and the importance of science in decision-making, reflected by the creation of an Intergovernmental Science Panel for the Amazon, a new collaborative effort that should contribute to the existing Science Panel for the Amazon, created by the UN Sustainable Solutions Network in 2019. Therefore, the implementation of this plan is highly dependent on scientific data supporting and guiding this concerted governmental action.

Within this context, it is key to foster scientific collaboration across the Amazonian countries on the entire spectrum of ecological and human health and well-being impacts. With this aim, the authors of this article organized a workshop on Mercury contamination and co-exposures on September 15, 2023, during the Annual Global Health Conference for the Americas, held in Cartagena, Colombia, and hosted by the Stempel College of Public Health and Social Work of Florida International University. The same workshop was held on November 29, 2023, at the Virtual Global Health Satellite Session of the Consortium of Universities for Global Health CUGH2024 [10]. The authors reported updates on metal exposure and health studies in the Amazonian regions of Colombia (JOV), Brazil (MECL, PCB), and Peru (MCG, CV, COG), as well as the overlapping impacts of microplastics (RL) and agrochemicals (AD). In recent times, increased deforestation in the Amazonian region to meet the demand for cash crops, mining, and many other anthropogenic activities have accelerated the use of agrochemicals, including fertilizers, pesticides, endocrine-disrupting chemicals, and POPs. This presents dangerous environmental health consequences for all Amazonian populations (urban and rural populations, traditional communities, and ~ 200 Indigenous populations, who live and work in this region). Exposures to agrochemicals, combined with mercury, cadmium, and lead-like heavy metals, have been shown to have placed the fragile ecosystem of the Amazonian region under threat due to increased loss of biodiversity and contaminated groundwater, air, and food supply chains.

The uniqueness of the Amazonian region and its relevance to planetary health warrant a precision environmental health approach to understand the impacts of exposure levels of heavy metals and agrochemicals, alone or in combination, on the biological, environmental, and social well-being of the populations and the ecosystem. The Amazonian regions, especially Brazil, are the major users of agrochemicals worldwide, with a billion liters of chemicals applied to crops, and this has a concerning repercussion in the Amazon basin, where studies are also targeting exposure levels and health outcomes in the population [11]. Studies show the presence of various agrochemicals in drinking water samples, above the LOD [12]. Despite this, current river basins like the Amazon lack uniform pesticide regulations, and the Amazonian countries should promote transboundary environmental management by collaborating on agrochemicals regulation establishment, aligned across environmental compartments, raising awareness of the toxicological impacts on human health [13].

The authors of this working group recognize the limitations of the lack of systematic studies conducted on the associations of heavy metals, including mercury and other heavy metals, and agrochemicals or their combinations thereof with neurodevelopmental, neurocognitive, neurodegenerative, and chronic disorders among the vulnerable populations of the Amazonian region. This lack is demonstrated, for example, in Brazilian official records of suspected/confirmed cases of mercury exposure/intoxication, showing most of these cases outside the Amazon [5]. There are challenges in conducting such studies to collect and analyze data on human populations, but the consortium of such experts from across the Amazonia will bring contextual knowledge and expertise to integrate local resources and interdisciplinary collaborations to bring out meaningful epidemiological associations of deleterious exposures, (individual or mixtures of pollutants) or determine causality of neurotoxicological and chronic disorders.

The authors consider the focus on mercury contamination and consequent human exposure and health impacts as the epiphenomenon of a more complex interaction between multiple factors, including agrochemicals, potentially affecting the ecosystem and human health. All interconnected factors affecting the Amazon basin must be considered with a holistic approach to provide scientific evidence and strategies suitable to effectively address the inequalities posed to the ethnically disadvantaged 1.6 million Indigenous people living in this region.

This article summarizes the updated occupational and environmental health findings in key areas of the Amazon and aims to foster the development of an academic partnership to strengthen epidemiological power and optimize laboratory capabilities, outreach, and community engagement resources. A stronger academic partnership will deliver more effective scientific evidence within the Science Panel for the Amazon of the UN Sustainable Solutions Network, and to the policymakers within the United Nations Framework Convention on Climate Change (UNFCCC) that will meet at the next Conference of the Parties (COP30) in Belém, Brazil in November 2025.

## 2. Materials and Methods

This review includes a summary of the most updated findings presented in the workshop on Mercury contamination and co-exposures by seven academic and governmental scientific centers, actively involved in this region: Grupo de Química Ambiental y Computacional, Universidad de Cartagena, Colombia (author JOV); Escola Nacional de Saúde Pública, Fundação Oswaldo Cruz - Fiocruz, Rio de Janeiro, Brazil (author PCB); Laboratorio de Farmacología Molecular, Universidade Federal do Pará (UFPA), Belém, Brazil (author MECL); Centro Nacional de Salud Ocupacional y Protección del Ambiente para la Salud. Instituto Nacional de Salud, Lima, Peru (author MCG); Centro de Innovación Científica Amazónica CINCIA, Puerto Maldonado, Peru (author CV); Universitat Oberta de Catalunya, and ISGlobal, Barcelona, Spain (author COCG); Universidad del Desarrollo, Las Condes, Chile (autor SMMB); Environmental and Global Health Sciences, Florida International University (authors RGL, AD, QF, CE).

## 3. Results

### Mercury fish contamination and human exposure

Table 1 lists the specific areas and local communities targeted by the studies conducted in the Amazonian areas of Colombia, Brazil, and Peru, showing the average level of mercury in hair and in fish.

**Table 1:** Mercury levels in fish and hair samples in Amazonian sites.

LOCATION, YEAR OF PUBLICATION, REFERENCE	POPULATIONS	FISH MERCURY LEVEL (AVERAGE ± SD IN µG/G)	HAIR MERCURY LEVEL* (AVERAGE ± SD IN µG/G)
Colombia			
Apaporis Natural Park, 2020 [14]	Yaigojé (Indigenous)		23.0 ± 1.2
Puerto Nariño, 2020 [15]			5.31 ± 0.49
Cotuhe Reservation, 2019 [16]	Tikuna (Indigenous)		10.6 ± 0.4
Caqueta River, 2016 [17]		0.10 ± 0.00 – 1.60 ± 0.30	15.35 ± 2.53 – 19.67 ± 1.63
Brazil			
Mucajaí River, Roraima, 2024 [18]	Yanomami adults (Indigenous)		3.9 ± 1.7
Mucajaí River, Roraima, 2024 [19]	Yanomami children (Indigenous)		3.7 (median)
Tapajós River, 2024 [20]	Munduruku (Indigenous)		5.5 (median)
17 locations (6 state capitols and 11 municipalities) of the Brazilian Amazon, 2023 [21]	Urban population	0.13 (median) 0.00–4.73 (range)	
Tucuruí Lake, 2023 [22]	Riverine populations		4.38 (2.35–6.78) – 15.5 (11.9–19.9)
Sawré Muybu Indigenous Land, 2021 [23, 24]	Munduruku (Indigenous)	0.10 ± 0.09 (non-piscivorous) 0.44 ± 0.34 (piscivorous)	7.7 ± 4.5
Paapiu region, Roraima, 2018 Waikás Ye’kuana region, Roraima, 2018 Waikás Aracaçá region, Roraima, 2018 [25]	Yanomami and Ye’kuana (women and children <5yrs Indigenous)		3.2 (median) range 0.4–8.6 4.5 (median) range 0.4–22.1 15.5 (median) range 4.6-20.4
Tapajós River, 2010 [26]	Riverine populations	0.77 ± 0.67 – 0.23 ± 0.17	4.1 ± 1.3 – 24.6 ± 17.8
Peru			
Manu River in Manu National Park, 2022 [27]	Matsigenka (Indigenous)		7.05 ± 05
Madre de Dios River, 2018 [28]		0.18 ± 0.15 – 0.28 ± 0.24	

(*): Compared to the reference doses of 1 µg/g or ppm for the US EPA and 1.6 µg/g or ppm for the WHO.

Piscivorous or carnivorous fish from gold-mining areas generally showed higher mercury concentrations, followed by fish from lower trophic levels, such as omnivorous, detritivores, and herbivorous species. Notably, riverside populations are highly frequent consumers of fish [29], causing a high intake of mercury. Besides fish, the consumption of Brazilian nuts and coconut milk [26], as well as other Amazonian fruits such as açaí [30] increases the dietary introduction of selenium, which may be a protective factor against mercury toxicity. Hair results are commonly utilized to monitor dietary exposure to methylmercury (MeHg) through fish consumption among the Amazonian communities. Occupational exposure to inorganic mercury, generated by inhalation of mercury vapors, is usually monitored through urine samples. Overall, the levels of mercury in human biological samples and in fish samples are overwhelmingly above the recommended reference standards by WHO, EPA, and international and national regulatory agencies. The exposure biomarkers are slightly higher in children, and in males compared to females. Very few studies have been conducted on Amazonian workers [31], showing that in the urine samples of the miners (named as garimpeiros), mercury levels ranged from 4 to 450 µg/L which is more reflective of their exposures to inorganic/elemental forms of mercury [26, 32]. In the Colombian Amazon, the studies led by Olivero-Verbel and colleagues have consistently reported elevated mercury levels, particularly among Indigenous groups in protected areas such as Yaigojé Apaporis. Findings revealed average hair concentrations exceeding 23 µg/g, way above the international thresholds, and resulted in associations with symptoms consistent with mercury-induced neurotoxicity, including sensory deficits and motor alterations [14].

Scarce reports are available from other Amazonian countries like Ecuador [33, 34] and Venezuela [35]. Average hair mercury levels of 27 µg/g, among the highest of the entire Amazonian basin, were measured in the Wapichana indigenous villages of the Marudi mountain region in Guyana [36], where the mining activities have been a common practice for more than a century and are further increasing after the Brazilian government cleared the Yanomami region from illegal mining in 2023, causing massive crossing of the Guyana border with miners and machineries.

### Health impacts of mercury exposure

A variety of 34 cross-sectional studies were published between 1996 and 2021, showing cognitive, vision, motor, somatosensory, and emotional deficits related to mercury body burden. These studies examined environmentally exposed adults (14) and children (16), with less focus on occupationally exposed workers (4) [31]. Additional studies have been published afterward and are included in this review, showing relevant aspects of cofactors, such as malnutrition, further affecting the most vulnerable groups of children and women of childbearing age [37], and reflected by high rates of stunting [38]. Mental health impacts including nervousness and irritability have also been observed in association with hair mercury [39]. Peripheral neurotoxicity associated with hair mercury has also been demonstrated among the Yanomami adults living in the northern Amazon [18]. Children of the same population have shown an inverse association of IQ with hair mercury levels [19], similarly to adults of the Matsigenka population in the Peruvian Amazon [27]. As a product of life-time exposure to mercury, clinical cases of neurological impacts are likely to be underestimated and underreported, despite the existing surveillance system of SINAN (*Sistema de Informação de Agravos de Notificação*) implemented in Brazil, in parallel to the community health workers program of ACS (*Agentes Comunitários de Saúde*) that coordinates public health data collection [5].

Besides the neurological hazards, an alarmingly high prevalence of chronic dysmetabolic conditions, including hypertension, insulin resistance, obesity, and atherogenic dyslipidemia, is present among the Amazonian riverine populations and at higher rates compared to the urban Amazonian groups [40]. Hair mercury results were cross-sectionally associated also with dyslipidemia and cardiovascular risk in the State of Pará, Brazil [22].

A highly relevant finding on genetic susceptibility has shown that the genotype APOE4 of apolipoprotein E is not only a risk factor for Alzheimer’s Disease, but also that APOE4 carriers, of Amerindian origin, can accumulate more mercury and are therefore at even higher neurological risk [41]. The tripeptide glutathione (GSH) can metabolize MeHg through its conjugation catalyzed by the glutathione S-transferases, particularly the pi1 isoform (GSTP1) that allows excretion via the ABC transporter system through the bile. Therefore, the presence of GSTP1 rs1695 has shown a similar influence, increasing mercury exposure measured in hair samples and impacting neurodevelopmental performance in small children from Indigenous groups [20] as well as among adults of the same villages [42].

### Co-Exposure to other metals, microplastics, and agrochemicals

Oil extraction in the Marañón, Pastaza, Tigre, and Corrientes River basins of the Peruvian Amazon generates high exposure to heavy metals. Among 1047 individuals from the local Indigenous populations, blood lead levels averaged 4.9 µg/dl (95%CI 4.5, 5.4) in individuals < 12 years and 5.7 µg/dl (95%CI 5.4, 6.) in older individuals [43]. Urinary arsenic averaged 3.61 µg/g creatinine as median (IQR 2.40) among males and 2.07 µg/g creatinine as median (IQR 2.07) among females; urinary cadmium averaged 1 µg/g creatinine as median (IQR 0.94) among males and 1.22 µg/g creatinine as median (IQR 0.88) among women [44].

According to the US National Oceanic and Atmospheric Administration (NOAA) and the European Chemicals Agency, microplastics are defined as fibers < 5 mm (0.20 in) in length. They are formed by carbon-based polymers that can include > 10,000 chemicals, such as phthalates, bisphenols, per-and poly-fluoroalkyl substances (PFAS), brominated and organophosphate flame retardants, and metals. They can leach into the environment with increasing temperature and light and are considered to cause neurotoxic, carcinogenic, immune- and endocrine-disrupting impacts on humans [45]. High bioaccumulation of nano- and microplastic fragments has been detected in the brain of deceased people in the USA, with the time of death (2016 versus 2024) being a significant factor indicating increasing concentrations over time, and even greater accumulation in the brains with dementia diagnosis, with particles deposited in cerebrovascular walls and immune cells [46]. Research on microplastic contamination and human exposure has started to take place in the Brazilian Amazon, with a first publication in 2018 [47]. A few studies have been conducted in these 7 years in different areas of the Amazon, showing a widespread presence of microplastics in water, sediments, biota, and fish [48].

Numerous studies have linked agrochemicals, including pesticide exposures, to adverse health outcomes in humans, particularly an elevated risk of various cancers [49]. In the last 10 years, Chlorpyrifos (pesticide), Malathion (pesticide), Atrazine (herbicide), Carbendazim (fungicide), and Mancozeb (fungicide) have been extensively used in the Amazonia to primarily support soy, maize, rice, sorghum, wheat, and cotton crops. The International Agency for Research on Cancer (IARC) also reported the evaluations of malathion and atrazine and listed them in groups 2A and 3, respectively, based on their different levels of carcinogenic potential [50]. Overall, pesticide consumption in the Amazon region has increased by 78% over the past decade [51]. In Brazil, a study examining the spread of soy cultivation found that agricultural pesticide exposure was associated with increased childhood cancer mortality. The researchers estimated that 123 childhood deaths during the 2008–2019 period were associated with exposure to pesticides from soy fields [52]. Brazil is the major user of agrochemicals worldwide, with billions of liters of chemicals applied to crops, and this has a concerning repercussion in the Amazon basin, where studies are also targeting exposure levels and health outcomes in the population [11]. Studies show the presence of various agrochemicals in drinking water samples above the LOD [12]. A systematic review found positive associations between pesticide exposure and several cancers, including non-Hodgkin lymphoma, leukemia, brain, and prostate cancers [53]. The review highlighted that many studies showed dose–response relationships, indicating that higher exposure levels correlate with increased cancer risk. Despite this, current river basins like the Amazon lack uniform pesticide regulations, and the Amazonian countries should promote transboundary environmental management, by collaborating on agrochemicals regulation establishment, aligned across environmental compartments, raising awareness of the toxicological impacts on human health [13].

## 4. Discussion

Despite the high risks of heavy metals and chemical contamination in the Amazon, and the dramatic repercussions on the natural biome and human health of workers and disadvantaged, malnourished, and vulnerable Indigenous and riverine populations, available scientific research is scarce, and relevant gaps of knowledge need urgently to be filled. Since the first publication in 1996 on mercury effects in the Brazilian Amazon, 34 studies have been published until 2021 [31] and about a dozen additional ones until today, which means that the scientific evidence is increasing, but still largely insufficient. Current research is mostly entirely based on cross-sectional studies and fragmented into a variety of sublocations in the Amazon basin. A clear need for more unified large cohort studies is envisaged to fill the current gap of knowledge on the longitudinal trajectories of exposures and related health impacts. Pooling together all participants in the 34 cross-sectional studies conducted until 2021 would generate a multi-age cohort of 7,105 individuals that may reach about 8,000, adding the most recent studies. Consortia of large cohort studies are highly productive in yielding more robust scientific evidence in occupational and environmental health, and, given the current situation of high concern at the international level, more science needs to be delivered also to policymakers.

All measurements of mercury contamination in fish and human biomarkers considered in our review show consistently higher levels compared to the recommended reference values in all study sites of the Amazon. The US EPA defined a reference dose (RfD) of 0.1 µg/kg bw/day corresponding to 1.0 µg MeHg/g [54] in hair samples. The FAO/WHO recommends doses of 1.6 µg/kg bw/week for the general population, corresponding to mercury hair levels of 2.3 µg/g, in hair samples [7]. These varying estimates of safe limits are caused by the current gap of knowledge on health impacts, especially considering the long-term effects of mercury exposure.

The hair values of the Amazonian studies are also significantly higher when compared to other recent studies conducted in countries like Japan and Italy. The Japanese research has found differences in brain structure at MRI assessment and impacted neurobehavioral and mood testing among 920 university students with high dietary fish intake, associated with hair mercury averaging 2.01 ± 1.15 µg/g among males and 1.85 ± 1.19 among females [55]. The Italian study showed an average of 1.63 ± 1.50 among 301 adults living in Trieste, the capital of the North-eastern region of the Adriatic Sea, with high mercury contamination caused by discharges from the cinnabar mining district of Idrija, Slovenia [56]. The hair levels of both studies from Japan and Italy were influenced, respectively, by high fish consumption and water contamination from mining operations. Overall, human exposure to mercury in the Amazon is currently among the highest in the world [57, 58]. Nevertheless, health impacts on the affected people remain underestimated.

Exposure levels to other highly toxic metals such as lead, arsenic, and cadmium are also exceeding the international reference standards and further increase the health risk of neurological, renal, cardiovascular, and carcinogenic impacts. Microplastics are an increasing additional threat to the Amazonian biome and human health, driven by the unregulated high urbanization, especially growing along the rivers’ margins, with poor sanitation conditions, translating into increasing contamination of the water and food chain [59]. Given their worldwide distribution and persistence in global water ecosystems, mercury and microplastics pose major threats to global water ecosystems. Experimental studies show that microplastic co-exposure can enhance mercury toxicity in a size-dependent manner through increased mercury accumulation either by serving as mercury carriers or by disrupting the xenobiotic resistance of organisms [60]. Microplastics enhance mercury toxicity mainly via increased oxidative apoptosis, compromised cell/organ morphogenesis, and energy depletion. Additionally, phosphor-proteomic analysis revealed impaired neuronal activity under combined exposure [61]. There are challenges in conducting such studies to collect, analyze, and integrate data on human populations, but the consortium of such experts from across the Amazonia will bring contextual knowledge and expertise to apply local resources and interdisciplinary collaborations to bring out meaningful epidemiological associations of deleterious exposures (individual or mixtures of pollutants) or determine causality of neurotoxicological and chronic disorders. The ecological implications are significant, as various organisms can ingest microplastics, leading to physical harm, reduced feeding, and impaired reproduction. In the Amazon, fish and other aquatic species have been found with microplastics in their digestive systems, which can disrupt food webs and potentially affect human populations that rely on these species for sustenance.

In addition to measuring the exposure levels of heavy metals and agrochemicals in the environment in the biological samples, this working group has extensively observed traditional environmental and occupational epidemiological associations between harmful exposures and neurotoxicological outcomes. Further, recent studies by the authors of this group (AD, QF) have used modern bioinformatic tools and machine-learning integrative approaches to convincingly demonstrate the associations of agrochemicals, heavy metals, and endocrine-disruptive chemical exposures with chronic disorders, including cancers [62, 63]; [64]. It has been suggested that interaction between the mixture of pesticides and the extreme climatic scenario further enhances the genotoxic and liver tissue damage in a fish model, in the latter case occurring even with the increase in the activity of antioxidant and biotransformation enzymes [65]. The authors (AD and QF) have used the Comparative Toxicogenomic Database in their studies to delineate functional and pathway data to aid in developing hypotheses about the mechanisms underlying environmentally influenced diseases.

Definitively, researchers, particularly those studying the Amazon’s ecosystems and its communities, bear a responsibility to: (i) approach their work in a way that is intellectually equitable, fostering inclusivity and respecting the contributions of local researchers and communities, as recently recommended [66] and (ii) make a greater effort to present their findings in ways that are clear and accessible to decision-makers of the Amazonian countries. This inclusivity is critical for the successful implementation of the Minamata Convention on Mercury, for example, which will depend on the backing of the entire Amazonian society (including urban dwellers, traditional communities, local researchers and professionals, and political figures).

Beyond direct health outcomes, the exposure to the mix of hazards that we have presented in this review also undermines cultural continuity by impairing elders’ ability to transfer ancestral knowledge, and limiting the participation of younger generations in traditional practices such as fishing and food preparation. This cultural disruption exacerbates the marginalization of Amazonian Indigenous peoples and deepens environmental injustice, making it a specific research issue that needs to be considered. To address these inequalities, the Oswaldo Cruz Foundation (Fiocruz), in collaboration with the Secretariat of Indigenous Health of the Ministry of Health (Sesai/MS) and the Ministry of Indigenous Peoples (MPI), introduced the Technical Manual for the Care of Indigenous People Exposed to Mercury in Brazil on April 30, 2025. This manual synthesizes current clinical protocols for the diagnosis and treatment of mercury intoxication while outlining healthcare pathways tailored to the sociocultural specificities of Indigenous communities. By emphasizing principles such as self-determination, human dignity, and the right to prior, free, and informed consultation, the document seeks to ensure that health services are delivered in a culturally sensitive and ethically grounded manner. Beyond its role as a clinical guide, the manual represents a pivotal step toward acknowledging the urgent need for integrated policies that safeguard Indigenous peoples’ rights to health, security, and environmental harmony within their ancestral territories [67].

## 5. Conclusions

We aim to increase awareness of the occupational and environmental health impacts of increasingly alarming heavy metal and chemical contamination in the Amazon basin produced by various non- or scarcely regulated, legal, and illegal anthropogenic operations and its impact on extreme climate conditions. Scientific evidence is urgently needed, not only for educational purposes for the local exposed populations but also to be delivered to the international policy level, namely the Intergovernmental Science Panel for the Amazon, a collaborative effort created in 2023 that should contribute to the existing Science Panel for the Amazon, created by the UN Sustainable Solutions Network.

Chemical contamination is increasingly occurring in the local and global environment, with multiple toxicants synergistically interacting to make them bioavailable and augmenting the risk of several adverse health outcomes. A more effective unifying epidemiological approach is strongly encouraged to provide solid statistical and integrative power of diverse data sources, optimize resources, improve scientific quality, and ultimately yield tools for a transboundary regulatory approach throughout the Amazonian basin. Given the Amazon’s ecological importance and biodiversity, targeted innovative efforts and inclusivity are essential to mitigate heavy metal, microplastic, and agrochemical contamination and protect both environmental and human health.

Finally, this working group indicates an urgent need for policy action. Strengthening regulations and enforcing compliance can significantly reduce the impact of mercury and co-exposures on the Amazon ecosystem and the population. It is imperative to implement comprehensive monitoring programs to assess chemical levels in water, soil, and biota as an essential pillar for informed decision-making. International and country cooperation is necessary to accomplish these goals. Countries in the Amazon basin should work together to create cohesive strategies for monitoring and remediation. Community Education Programs should also be further developed, informing communities about the dangers of chemical exposures and safe fishing practices. In addition, raising awareness about the impacts of these contaminants on health and the environment can mobilize public support for policy changes and sustainable practices. Efforts to mitigate mercury impacts must center not only on exposure reduction, but also on principles of environmental justice and equitable science, ensuring that indigenous voices are not just included, but drive the solutions. This is particularly urgent considering that mercury pollution is being driven by the global demand for gold—a metal with limited practical use, whose primary function is to accumulate wealth in banks and vaults. Entire ecosystems and Indigenous populations are being sacrificed for a commodity that offers no essential benefit to humanity, raising profound ethical questions about extractive economies and the value we place on life and ecosystems. Addressing these challenges in the Amazon requires a multi-stakeholder approach that combines strong policy action with comprehensive educational efforts.
